# Molybdate toxicity in Chinese cabbage is not the direct consequence of changes in sulphur metabolism

**DOI:** 10.1111/plb.13065

**Published:** 2019-12-06

**Authors:** E. I. Zuidersma, T. Ausma, C. E. E. Stuiver, D. H. Prajapati, M. J. Hawkesford, L. J. De Kok

**Affiliations:** ^1^ Isotope Laboratory Life Sciences Graduate School of Science and Engineering University of Groningen Groningen The Netherlands; ^2^ Laboratory of Plant Physiology Groningen Institute for Evolutionary Life Sciences University of Groningen Groningen The Netherlands; ^3^ Department of Biotechnology Hemchandracharya North Gujarat University Patan Gujarat India; ^4^ Plant Sciences Department Rothamsted Research Harpenden UK

**Keywords:** *Brassica*, heavy metals, molybdenum, sulphate assimilation, sulphate uptake

## Abstract

- In polluted areas, plants may be exposed to supra‐optimal levels of the micronutrient molybdenum. The physiological basis of molybdenum phytotoxicity is poorly understood. Plants take up molybdenum as molybdate, which is a structural analogue of sulphate. Therefore, it is presumed that elevated molybdate concentrations may hamper the uptake and subsequent metabolism of sulphate, which may induce sulphur deficiency.
- In the current research, Chinese cabbage (*Brassica pekinensis*) seedlings were exposed to 50, 100, 150 and 200 μm Na_2_MoO_4_ for 9 days.
- Leaf chlorosis and a decreased plant growth occurred at concentrations ≥100 μm. Root growth was more affected than shoot growth. At ≥100 μm Na_2_MoO_4_, the sulphate uptake rate and capacity were increased, although only when expressed on a root fresh weight basis. When expressed on a whole plant fresh weight basis, which corrects for the impact of molybdate on the shoot‐to‐root ratio, the sulphate uptake rate and capacity remained unaffected. Molybdate concentrations ≥100 μm altered the mineral nutrient composition of plant tissues, although the levels of sulphur metabolites (sulphate, water‐soluble non‐protein thiols and total sulphur) were not altered. Moreover, the levels of nitrogen metabolites (nitrate, amino acids, proteins and total nitrogen), which are generally strongly affected by sulphate deprivation, were not affected. The root water‐soluble non‐protein thiol content was increased, and the tissue nitrate levels decreased, only at 200 μm Na_2_MoO_4_.
- Evidently, molybdenum toxicity in Chinese cabbage was not due to the direct interference of molybdate with the uptake and subsequent metabolism of sulphate.

## Graphical abstract

Exposure to excessive molybdate may constrain plant growth. It is presumed that elevated molybdate concentrations may hamper the uptake and subsequent metabolism of sulfate, which may induce sulfur deficiency. However, our research showed that in Chinese cabbage molybdate toxicity was not due to the direct interference of molybdate with the uptake and subsequent metabolism of sulfate.

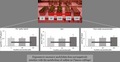

## Introduction

Molybdenum (Mo) is an essential micronutrient for plant growth, whose requirement is the lowest of all essential nutrients (Mendel, 2011). In most plant tissues Mo levels range between 2 and 20 nmol·g^−1^ dry weight (Hamlin, 2006). Molybdenum is predominantly present in soils in the form of molybdate (MoO_4_^2−^), which is also the main Mo source for plant growth (Hamlin, 2006; Bittner, 2014). Inside plants, molybdate may be bound to pterin, thereby forming the molybdenum cofactor (Moco; Mendel, 2011; Bittner, 2014). Upon incorporation of Moco as prosthetic group in molybdo‐enzymes, Mo is involved in metabolic redox reactions (Mendel, 2011; Bittner, 2014). Plants contain at least five different molybdo‐enzymes: nitrate reductase, sulphite oxidase, xanthine dehydrogenase, aldehyde oxidase and the mitochondrial amidoxime reductase (Bittner, 2014).

The uptake of molybdate by the root is presumably facilitated by distinct transporters. Two transporters, MOT1 and MOT2, have been identified to transport molybdate with high affinity (nanomolar *K*_m_ range; Buchner *et al.,* 2004; Tejada‐Jiménez *et al.,* 2007; Tomatsu *et al.,* 2007; Gasber *et al.,* 2011). Whereas MOT1 is predominantly expressed in root cells and involved in the uptake of molybdate into the plant, MOT2 is expressed in leaf tonoplast membranes and involved in vacuolar molybdate export (Tejada‐Jiménez *et al.,* 2007; Tomatsu *et al.,* 2007; Gasber *et al.,* 2011). Notably, both molybdate transporters highly resemble sulphate transporters (Buchner *et al.,* 2004; Bittner, 2014). Molybdate is structurally an analogue to sulphate (*viz.* both ions possess a double negative charge, are similar in size and have tetrahedral structures), reflecting the similarity of molybdate and sulphate transporters (Tomatsu *et al.,* 2007; Gasber *et al.,* 2011). Due to the structural analogy between molybdate and sulphate, it has been suggested that sulphate transporters can also transport molybdate. Accordingly, expression of the sulphate transporter SHST1 from Caribbean stylo (*Stylosanthes hamata*) in a yeast (*Saccharomyces cerevisiae*) mutant defective in sulphate transport, resulted in an increased capacity to take up molybdate (Fitzpatrick *et al.,* 2008). Furthermore, tissue molybdate levels are generally enhanced when plants are deprived of sulphur, which has been explained by an increased activity of the sulphate transporters upon sulphur deprivation (Schinmachi *et al.,* 2010; Schiavon *et al.,* 2012; Reich *et al.,* 2016).

Although Mo is an essential micronutrient, exposure to excessively high molybdate levels may inhibit plant growth (Xu *et al.,* 2018a,b). Soil molybdate concentrations can increase to supra‐optimal levels in agricultural soils due to industrial activities (*e.g*. mining; Gupta, 1997). The physiological basis for the phytotoxicity of molybdate remains elusive. Being a heavy metal, exposure to elevated molybdate concentrations may potentially inhibit the uptake of other essential metals (Pilon *et al.,* 2009; Cuypers *et al.,* 2009; Yadav, 2010). However, molybdate toxicity may also arise from the structural analogy between molybdate and sulphate: it is presumed that exposure to elevated molybdate levels may negatively affect sulphur metabolism in plants (Wangeline *et al.,* 2004; Fitzpatrick *et al.,* 2008). It may hamper sulphate uptake and transport. Since sulphate transporters are capable of molybdate transport, molybdate and sulphate may compete for the binding of the same transporter (Fitzpatrick *et al.,* 2008; Schiavon *et al.,* 2012). Accordingly, treatment with 25 µm molybdate down‐regulated the import of sulphate through the sulphate transporter SHST1 from Caribbean stylo in a yeast mutant defective in sulphate transport (Fitzpatrick *et al.,* 2008). Furthermore, exposure to 200 µm molybdate rapidly (*viz.* within 10 min) decreased sulphate import into the roots of brown mustard (*Brassica juncea*; Schiavon *et al.,* 2012). Molybdate exposure may also negatively affect sulphate metabolism in the chloroplast. The first enzyme of sulphate metabolism, ATP sulphurylase, can utilise molybdate instead of sulphate as its substrate, which may strongly inhibit the reduction of sulphate and its subsequent assimilation in cysteine and other organic sulphur compounds (Reuveny, 1977; Wangeline *et al.,* 2004). A 1‐day exposure of brown mustard to 200 µm molybdate decreased the levels of cysteine and glutathione to the same extent as exposure to sulphur deprivation (Schiavon *et al.,* 2012).

To obtain further insights into the significance of the interaction between molybdenum and sulphur metabolism for the phytotoxicity of molybdate, seedlings of Chinese cabbage (*Brassica pekinensis*) were exposed to elevated molybdate levels in the root environment for 9 days, and the impacts on the uptake and subsequent metabolism of sulphate were evaluated.

## Material and methods

### Plant material and growth conditions

Seeds of Chinese cabbage (*Brassica pekinensis* (Lour.) Rupr. cv. Kasumi F1 (Nickerson‐Zwaan, Made, The Netherlands) were germinated in vermiculite in a climate‐controlled room. Day and night temperatures were 22 and 18 °C (±1 °C), respectively, relative humidity was 60–70% and the photoperiod was 14 h at a photon fluence rate of 300 ± 20 µmol·m^–2^·s^–1^ (within the 400–700 nm range) at plant height, supplied by Philips GreenPower LED (deep red/white 120) production modules. After 11 days, seedlings were transferred to aerated 25% Hoagland nutrient solutions (pH 5.9). Sulphate concentration in the solution was 500 μm and Na_2_MoO_4_ concentration was 0.13 μm (for further details on the composition, see Shahbaz *et al.,* 2013). For the measurement of plant growth parameters and pigment content, plants were grown for 9 days on 13 l stainless steel containers (ten sets of plants per container, three plants per set), containing the nutrient solution with supplemental concentrations of either 0, 50, 100, 150 or 200 μm Na_2_MoO_4_. For the measurement of other parameters, plants were grown for 9 days on 30 l containers (ten sets of plants per container, three plants per set), containing the nutrient solution with additional Na_2_MoO_4_ concentrations of either 0, 100 or 200 μm.

### Growth analyses

After exposure, plants were harvested 3 h after the onset of the light period. To remove ions and other particles attached to the root, plant roots were rinsed in ice‐cold de‐mineralised water (3 × 20 s). Subsequently, shoots and roots were separated and weighed. Shoot and root biomass production were calculated by subtracting the initial, pre‐exposure, weight from the weight at the harvest. Additionally, shoot‐to‐root biomass ratio was calculated from the fresh shoot and root weights at harvest. For the determination of dry matter content, plant material was dried at 80 °C for 24 h.

### Chemical analyses

Chlorophyll content was determined in shoots, which were stored at −20 °C after harvest, according to Lichtenthaler (1987). The content of Mo and other elements was analysed in dried pulverised plant material *via* inductively coupled plasma optical emission spectroscopy (ICP‐OES) as described by Shahbaz *et al. *(2010). Total sulphur content was additionally determined with the barium sulphate precipitation method (Koralewska *et al.,* 2008). Total nitrogen content was determined according to a modified Kjeldahl method (Barneix *et al.,* 1988). Sulphate and nitrate levels were determined in plant material, which was stored at −20 °C after harvest, *via* high‐performance liquid chromatography (HPLC; Maas *et al.,* 1986). Water‐soluble non‐protein thiols were extracted from freshly harvested plant tissue and the total water‐soluble non‐protein content was determined colorimetrically according to De Kok *et al. *(1988). Water‐soluble proteins were extracted from −20 °C frozen plant tissue and determined colorimetrically by the method of Bradford (1976). Free amino acids were extracted similarly to sulphate and nitrate. Their content was determined *via* colorimetric determination of the ninhydrin‐reactive groups according to Stuiver *et al. *(1992). To assess the nitrate reductase activity of plant material, nitrate reductase was extracted from freshly harvested shoots or roots and the *in vitro* activity was analysed according to De Kok *et al. *(1986).

### Sulphate uptake

For the measurement of the sulphate uptake rate, plants grown for 8 days in the presence of different molybdate concentrations, were transferred to plastic beakers containing aerated 25% Hoagland solutions with identical molybdate concentrations as plants were grown on. Plants were incubated on these solutions for 24 h. Sulphate uptake rate was subsequently assessed following Westerman *et al. *(2000). For the determination of the sulphate uptake capacity (*viz.* the activity of the sulphate transporters), plants grown for 9 days in the presence of different molybdate concentrations were transferred to an aerated 25% Hoagland solution containing 500 µm ^35^S‐sulphate (2 MBq·l^−1^). This solution either contained an identical molybdate concentration as the plants were grown on or no supplemental molybdate. Plants were incubated on the solution for 30 min. Sulphate uptake capacity was then measured as outlined by Koralewska *et al. *(2007).

### Statistical analyses

Statistical analyses were performed using GraphPad Prism (GraphPad Software, San Diego, CA, USA). To compare treatment means an one‐way anova with a Tukey’s HSD test as *post‐hoc* test at the *P* ≤ 0.05 level was performed.

## Results and discussion

**Figure 1:**
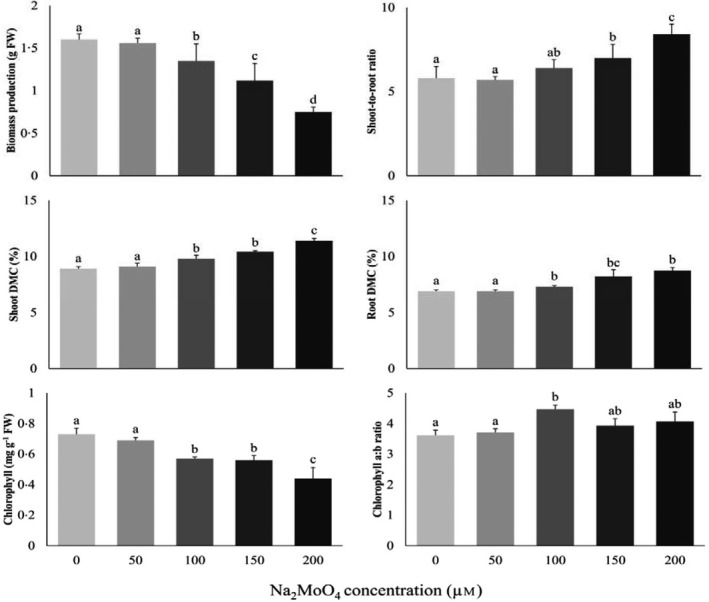
Impact of Na_2_MoO_4_ exposure on the growth of Chinese cabbage. 11‐day‐old seedlings were grown on a 25% Hoagland nutrient solution containing additional Na_2_MoO_4_ concentrations ranging from 50 to 200 µm for 9 days. The initial plant weight was 0.045 ± 0.005 g. Data on biomass production (g FW) and shoot‐to‐root ratio represent the mean of ten measurements with three plants in each (±SD). Data on dry matter content (DMC; %) and chlorophyll content (mg·g^−1^ FW) represent the mean of three measurements with three plants each (±SD). Different letters indicate significant differences between treatments (*P* < 0.05, one‐way anova; Tukey’s HSD test as a *post‐hoc* test).

A 9‐day exposure of Chinese cabbage to elevated molybdate levels significantly decreased biomass production at concentrations ≥100 µm Na_2_MoO_4_ (Fig. 1). Whereas at 100 µm biomass production was reduced with 15%, at 200 µm biomass production was more than 50% lowered. In line with previous observations (Schiavon *et al.,* 2012), root biomass production was more reduced than shoot biomass production, causing an increased shoot‐to‐root ratio (up to 1.4‐fold at 200 µm; Fig. 1). Notably, whereas exposure to excessive copper (Cu) inhibited root growth more than shoot growth, exposure to excessive zinc (Zn) and manganese (Mn) had the opposite impact (Shahbaz *et al.,* 2010, 2013; Stuiver *et al.,* 2014; Neves *et al.,* 2017). Similarly to other metals, the decreases in plant growth upon molybdate exposure were associated with increases in dry matter content in both shoot and root (up to 1.3‐fold at 200 µm; Fig. 1) and with leaf chlorosis, resulting in a strong decrease in the shoot chlorophyll content (up to 40% lower at 200 µm; Fig. 1). Nevertheless, the ratio between chlorophyll *a* and *b* remained unaffected (Fig. 1). The chlorosis upon excessive Mo exposure could be the result of an inhibited development of new chloroplasts, rather than the malfunctioning of existing chloroplasts, similar to observations in *Brassica* upon exposure to toxic Cu, Zn and Mn levels (Shahbaz *et al.,* 2010; Neves *et al.,* 2017).

The Mo concentration in Chinese cabbage increased with the level of Mo in the root environment (Table 1). However, the level of increase was higher in the shoot than in the root. Whereas at 100 µm Na_2_MoO_4_ shoot Mo levels increased 468‐fold (to 18.7 µmol·g^−1^ dry weight), root levels increased 80‐fold (to 35.3 µmol·g^−1^ dry weight; Table 1). Apparently, when tissue Mo levels exceeded these levels, it became rapidly phytotoxic for Chinese cabbage. The measured Mo toxicity values are in agreement with earlier reported values (Nautiyal & Chatterjee, 2004; Nie *et al.,* 2007; Schiavon *et al.,* 2012; Xu *et al.,* 2018a,b). However, the values are much higher than the toxicity values observed for other heavy metals: Cu, Zn and Mn were already toxic when tissue levels increased <10‐fold (Shahbaz *et al.,* 2010, 2013; Stuiver *et al.,* 2014; Neves *et al.,* 2017). Evidently, Chinese cabbage is relatively tolerant to elevated Mo levels.

**Table 1:** Impact of Na_2_MoO_4_ exposure on the tissue elemental composition of Chinese cabbage. 11‐day‐old seedlings were grown on a 25% Hoagland nutrient solution containing additional 0, 100 and 200 µm Na_2_MoO_4_ for 9 days.

Element concentrations	Na_2_MoO_4_ concentration (µm)		
(µmol·g^−1^ dry weight)	0	100	200
Shoot			
Calcium	723 ± 15a	699 ± 21a	537 ± 18b
Copper	0.17 ± 0.03a	0.18 ± 0.03a	0.13 ± 0.01a
Iron	1.41 ± 0.06a	1.22 ± 0.08a	0.86 ± 0.13b
Magnesium	178 ± 7a	182 ± 5a	157 ± 2b
Manganese	2.2 ± 0.1a	2.1 ± 0.1a	1.6 ± 0.1b
Molybdenum	0.04 ± 0.00a	18.7 ± 0.8b	38.4 ± 2.2c
Phosphorus	197 ± 2a	188 ± 7a	148 ± 2b
Potassium	1605 ± 42a	1605 ± 29a	1241 ± 42b
Sodium	14.9 ± 1.8a	36.4 ± 1.6b	54.6 ± 0.4c
Sulfur	231 ± 8a	226 ± 10a	239 ± 8a
Zinc	0.86 ± 0.29a	0.85 ± 0.07a	0.79 ± 0.07a
Root			
Calcium	189 ± 3a	393 ± 40b	453 ± 24b
Copper	0.49 ± 0.03a	0.55 ± 0.02ab	0.64 ± 0.07b
Iron	27 ± 1a	66 ± 5544a	34 ± 6a
Magnesium	153 ± 4a	150 ± 7a	131 ± 17a
Manganese	31 ± 3a	37 ± 2a	43 ± 8a
Molybdenum	0.44 ± 0.22a	35.3 ± 0.8b	58.6 ± 22.0b
Phosphorus	309 ± 7a	322 ± 5a	325 ± 8a
Potassium	1564 ± 15a	1383 ± 20b	1259 ± 108b
Sodium	17 ± 3a	26 ± 1b	29 ± 5b
Sulfur	333 ± 17a	305 ± 8a	305 ± 7a
Zinc	0.98 ± 0.12a	1.69 ± 0.15b	1.77 ± 0.36b

Data (µmol·g^−1^ DW) represent the mean of three measurements with nine plants in each (±SD). Different letters indicate significant differences between treatments (*P* < 0.05, one‐way anova; Tukey’s HSD test as a *post‐hoc* test).

Analogously to other heavy metals, Mo exposure may inhibit the uptake of other essential metals (Cuypers *et al.,* 2009; Pilon *et al.,* 2009; Yadav, 2010). However, it is doubtful if an inhibited metal uptake is the direct cause of Mo phytotoxicity in Chinese cabbage. Although exposure to 100 µm Na_2_MoO_4_ reduced plant growth, it increased the root content of calcium (Ca) and Zn (2.1‐fold and 1.7‐fold at 100 µm and 2.4‐fold and 1.8‐fold at 200 µm, respectively; Table 1). Furthermore, although it decreased root potassium (K) levels (12% at 100 µm and 20% at 200 µm, respectively; Table 1), this decrease may also be related to the enhanced tissue sodium (Na) levels upon Na_2_MoO_4_ exposure: Na influx into roots may reduce the root influx of K (Koevoets *et al.,* 2016). Finally, exposure to 100 µm Na_2_MoO_4_ did not affect the elemental composition of shoots (apart from the Mo and Na content; Table 1). The elemental composition of the shoot was only affected upon exposure to 200 µm Na_2_MoO_4_, which decreased the contents of Ca, iron (Fe), magnesium (Mg), Mn, phosphorus (P) and K with 26%, 39%, 12%, 27%, 25% and 23%, respectively (Table 1).

Molybdenum phytotoxicity may also arise from the structural resemblance between molybdate and sulphate (Reuveny, 1977; Wangeline *et al.,* 2004). Exposure to elevated molybdate concentrations may hamper the uptake and subsequent metabolism of sulphate in plants. Exposure of brown mustard to 200 µm molybdate rapidly (within 10 min) down‐regulated the root influx of sulphate and after 1 day, had significantly decreased the content of water‐soluble non‐protein thiols (cysteine and glutathione; Schiavon *et al.,* 2012). However, in contrast, exposure of Chinese cabbage seedlings for a prolonged period (9 days) to high molybdate levels did not negatively affect sulphate uptake and subsequent metabolism. It was evident that exposure to ≥100 µM Na_2_MoO_4_ enhanced the sulphate uptake rate (measured over the last 24 h of exposure), although only when this rate was expressed on a root fresh weight basis (*viz.* per gram root; Table 2). When expressed on a whole plant fresh weight basis (*viz.* per gram plant, which takes changes in shoot‐to‐root ratio into account), the rate remained unaffected (Table 2). Similarly, there was an increase in sulphate uptake capacity (*viz.* the activity of the sulphate transporters), but again only when expressed on a root fresh weight basis (Table 2). The sulphate uptake capacity was not affected by the presence or absence of supplemental Na_2_MoO_4_ during the uptake capacity measurements (Table 2). Evidently, there was no direct competition between the uptake of sulphate and molybdate by Sultr1;2, which is the high affinity sulphate transporter mainly responsible for the uptake of sulphate by *Brassica* roots at sulphate‐sufficient conditions (Koralewska *et al.,* 2007, 2008).

**Table 2:** Impact of Na_2_MoO_4_ exposure on the sulphate uptake rate and capacity of Chinese cabbage. 11‐day‐old seedlings were grown on a 25% Hoagland nutrient solution containing additional 0, 100 and 200 µm Na_2_MoO_4_ for 8 (sulphate uptake rate) or 9 days (sulphate uptake capacity).

	Na_2_MoO_4_ concentration (µm)		
	0	100	200
uptake rate			
root basis	1.59 ± 0.10a	2.13 ± 0.12b	3.34 ± 0.34c
plant basis	0.26 ± 0.02a	0.32 ± 0.02a	0.35 ± 0.08a
uptake capacity			
root basis			
−MoO_4_^2−^	1.56 ± 0.07a	1.85 ± 0.11b	3.06 ± 0.03c
+MoO_4_^2−^		1.85 ± 0.09b	3.15 ± 0.57c
plant basis			
−MoO_4_^2−^	0.268 ± 0.015a	0.264 ± 0.027a	0.311 ± 0.026a
+MoO_4_^2−^		0.263 ± 0.010a	0.314 ± 0.052a

Sulphate uptake rate (µmol·g^−1^ FW·h^−1^) was measured over a 24‐h period after transferring plants to fresh nutrient solutions with an identical Na_2_MoO_4_ level as that on which the plants were grown. Sulphate uptake capacity (µmol·g^−1^ FW·h^−1^) was measured over a 30‐min period on a ^35^SO_4_^2−^‐labelled 25% Hoagland nutrient solution, which either contained an identical molybdate concentration as that on which the plants were grown or no supplemental molybdate. Data represent the mean of four measurements with three plants in each (±SD). Different letters indicate significant differences between treatments (*P* < 0.05, one‐way anova; Tukey’s HSD test as a *post‐hoc* test).

Additionally, levels of sulphur metabolites were not affected upon exposure to 100 µm Na_2_MoO_4_. The sulphate and water‐soluble non‐protein thiol content in both roots and shoots remained unaltered (Table 3). Consequently, the total sulphur content, measured with the barium sulphate precipitation method (Table 3) and with the ICP‐OES method (Table 1) remained unaltered. Exposure to 200 µm Na_2_MoO_4_ did also not affect the sulphate and total sulphur content of plants (Table 3). However, by contrast, this treatment enhanced the water‐soluble non‐protein thiol content of roots approximately 1.5‐fold (Table 3).

**Table 3:** Impact of Na_2_MoO_4_ exposure on the sulphur and nitrogen metabolism of Chinese cabbage. 11‐day‐old seedlings were grown on a 25% Hoagland nutrient solution containing additional 0, 100 and 200 µm Na_2_MoO_4_ for 9 days.

	Na_2_MoO_4_ concentration (µm)		
	0	100	200
Shoot			
Sulphate	11.6 ± 0.9a	10.6 ± 0.4a	13.0 ± 2.9a
Thiols	0.51 ± 0.08a	0.57 ± 0.05a	0.59 ± 0.03a
Total sulphur	0.216 ± 0.001a	0.212 ± 0.004a	0.214 ± 0.009a
Nitrate	54.3 ± 3.7a	49.5 ± 5.7a	22.2 ± 8.5b
Amino acids	19.9 ± 5.3a	19.2 ± 2.5a	21.1 ± 3.1a
Proteins	10.1 ± 0.2a	9.9 ± 0.2a	9.3 ± 1.2a
Total nitrogen	4.33 ± 0.06b	4.22 ± 0.17b	2.89 ± 0.07a
NR activity	10.4 ± 1.4a	11.7 ± 0.3a	10.3 ± 0.9a
N/S ratio	20.0 ± 0.3b	19.9 ± 1.2b	13.4 ± 0.9a
Root			
Sulphate	11.8 ± 0.4a	11.1 ± 0.6a	11.5 ± 0.9a
Thiols	0.47 ± 0.03a	0.52 ± 0.03a	0.68 ± 0.08b
Total sulphur	0.296 ± 0.011a	0.286 ± 0.025a	0.307 ± 0.015a
Nitrate	45.9 ± 3.7a	43.0 ± 2.3a	24.0 ± 2.0b
Amino acids	17.7 ± 2.8a	20.7 ± 2.0a	21.8 ± 3.2a
Proteins	5.0 ± 0.5a	5.5 ± 0.3a	6.1 ± 0.4a
Total nitrogen	4.10 ± 0.02a	4.11 ± 0.05a	3.65 ± 0.09b
NR activity	1.8 ± 0.7a	1.8 ± 0.5a	1.9 ± 1.2a
N/S ratio	13.9 ± 0.6a	14.3 ± 1.4a	11.9 ± 0.9b

Data on sulphate, water‐soluble non‐protein thiols, nitrate, free amino acids (µmol·g^−1^ FW), water‐soluble proteins (mg·g^−1^ FW) and *in vitro* nitrate reductase activity (µmol·g^−1^ FW·h^−1^) are the mean of two experiments with three measurements on three plants in each (±SD). Data on total sulphur and nitrogen (mmol·g^−1^ DW) represent the mean of three measurements on 18–24 plants from two pooled experiments (±SD). Different letters indicate significant differences between treatments (*P* < 0.05, one‐way anova; Tukey’s HSD test as a *post*‐*hoc* test).

The nature of the root thiol accumulation needs further evaluation. It may be attributed to changes in the levels of cysteine, glutathione and/or phytochelatins (Cuypers *et al.,* 2009). Glutathione has antioxidant capacities and consequently may protect plants against heavy metal stress (Cuypers *et al.,* 2009). Phytochelatins are small peptides which can bind and sequestrate metals (Cuypers *et al.,* 2009). Notably however, exposure to excessive Mn did not affect the water‐soluble non‐protein thiol content of tissues (Neves *et al.,* 2017). Furthermore, although exposure to excessive Zn and Cu enhanced the water‐soluble non‐protein thiol content of tissues, it was doubtful if this increase had physiological significance for the detoxification of the heavy metal: experimental manipulation of the size and composition of the thiol pool did not affect the Cu tolerance of Chinese cabbage (Shahbaz *et al.,* 2013). The thiol pool was more likely altered as the consequence of a deregulated thiol metabolism in the presence of excessive Cu (Shahbaz *et al.,* 2013).

Nitrogen metabolism, which is typically profoundly affected by sulphate deprivation (Hawkesford & De Kok, 2006), was hardly impacted by exposure to 100 µm Na_2_MoO_4_ (Table 3). In both root and shoot, the contents of nitrate, amino acids and proteins were unaffected (Table 3). Accordingly, the total nitrogen content and the activity of nitrate reductase remained unchanged (Table 3). Exposure to 200 µm Na_2_MoO_4,_ in contrast, affected nitrogen metabolism: it decreased the nitrate content in both shoot and root (60% and 48%, respectively; Table 3). Concomitantly, it decreased the total nitrogen content of the shoot and root. Notably, however, sulphur deprivation generally enhances tissue nitrate levels (Hawkesford & De Kok, 2006). Since the total sulphur content was not affected at 200 µm Na_2_MoO_4_, the decrease in total nitrogen content caused a drop in the nitrogen‐to‐sulphur ratio: 33% and 14% for the shoot and root, respectively (Table 3).

## Conclusions

Chinese cabbage seedlings were susceptible to elevated molybdate concentrations in the root environment when concentrations exceeded 100 µm. The phytotoxicity of molybdate did not directly arise from the chemical resemblance between molybdate and sulphate: a 9‐day exposure of Chinese cabbage seedlings to toxically high molybdate levels did not negatively interfere with the uptake and metabolism of sulphate.
